# Biomarker driven treatment of head and neck squamous cell cancer

**DOI:** 10.1186/s41199-017-0025-1

**Published:** 2017-08-29

**Authors:** Nnamdi Eze, Ying-Chun Lo, Barbara Burtness

**Affiliations:** 1 Section of Medical Oncology, Department of Internal Medicine, Yale University School of Medicine and Yale Cancer Center, 333 Cedar Street, Room WWW-221, P.O. Box 208028, New Haven, CT 06520 USA; 2 Department of Pathology, Yale University School of Medicine, New Haven, CT USA; 3 Section of Medical Oncology, Department of Internal Medicine, Yale University School of Medicine and Yale Cancer Center, New Haven, CT USA

**Keywords:** Head and neck squamous cell cancer (HNSCC), Biomarkers, Human papilloma virus (HPV), Epidermal growth factor receptor (EGFR), Epstein Barr virus (EBV), Cetuximab, Nasopharyngeal carcinoma (NPC), Phosphatase and tensin homolog (PTEN), Phosphoinositide 3- kinase (PI3K)

## Abstract

**Abstract:**

Treatment modalities of head and neck squamous cell cancer include surgery, radiation, chemotherapy, targeted agents and immune checkpoint inhibition. Treatment is often toxic and can affect long-term function and quality of life. In this context, identification of biomarker data that can help tailor therapy on an individualized basis and reduce treatment-related toxicity would be highly beneficial. A variety of predictive biomarkers have been discovered and are already utilized in clinical practice, while many more are being explored. We will review p16 overexpression as a surrogate biomarker in HPV-associated head and neck cancer and plasma EBV DNA as a biomarker in nasopharyngeal carcinoma, the two established biomarkers currently utilized in clinical practice. We will also examine novel predictive biomarkers that are in clinical development and may shape the future landscape of targeted head and neck cancer therapy. These emerging biomarkers include the tyrosine kinases and their signaling pathway, immune checkpoint biomarkers, tumor suppressor abnormalities, and molecular predictors of hypoxia-targeted therapy. We will also look at futuristic biomarkers including detection of circulating DNA from clinical specimens and rapid tumor profiling. We will highlight the ongoing effort that will see a shift from prognostic to predictive biomarker development in head and neck cancer with the goal of delivering individualized cancer therapy.

**Trial registration:**

N/A.

## Background

Head and neck squamous cell cancer (HNSCC) is a heterogeneous group of cancers accounting for about 3% of all cancers in the United States. Each year, an estimated 61,000 people develop HNSCC, of whom about 13,000 die [1]. Treatment modalities include surgery, radiation, chemotherapy, targeted agents and immune checkpoint inhibition. For the many patients who are cured, late sequelae of treatment can affect function, quality of life and possibly even non-cancer mortality [2–4]. In this context, indicators of biologic behavior and treatment sensitivity could prove enormously helpful in tailoring therapy on an individualized basis. This is the rationale behind the search for predictive and prognostic biomarkers in HNSCC. The National Cancer Institute (NCI) defines a biomarker as “a biological molecule found in blood, other body fluids, or tissues that is a sign of a normal or abnormal process or of a condition or disease; and may be used to see how well the body responds to a treatment for a disease or condition” [5]. Although biomarkers of Human Papilloma Virus (HPV) association have emerged as validated, standard biomarkers in this disease, numerous studies point to the potential utility of biomarkers in predicting outcome and selecting therapy. This review focuses on prognostic and predictive biomarkers that drive therapeutic choices in HNSCC. We will look at established biomarkers that are standard of care in clinical practice, as well as novel biomarkers that are in clinical development.

### Established biomarkers

With the identification of HPV as an etiologic agent in a subset of HNSCC, p16 overexpression by immunohistochemistry (IHC) as a surrogate marker of HPV association has become the most robust HNSCC biomarker employed in clinical practice. Plasma Epstein Barr Virus (EBV) Deoxyribonucleic Acid (DNA) also plays a role as a predictive and prognostic biomarker specifically in nasopharyngeal carcinoma (NPC) patients.

### HPV status in oropharyngeal SCC (OPSCC)

HPV-initiated HNSCC is a biologically distinct category of HNSCC with significantly better prognosis and treatment outcome compared to HPV-negative HNSCC [6–8]. p16 overexpression by IHC is an outstanding surrogate marker of HPV association in OPSCC [9] and is well established as a prognostic biomarker of favorable outcome in HNSCC. p16, a tumor suppressor protein that is encoded by *CDKN2A gene*, regulates cell cycle by inhibiting the phosphorylation of the retinoblastoma (Rb) tumor suppressor protein by cyclin dependent kinases (CDK) 4 and 6. This leads to inactivation of factor E2F1, an important component of cell-cycle progression. In the setting of HPV-associated tumors, the HPV E7 viral oncoprotein promotes rapid degradation of Rb, and as Rb usually regulates p16, the disruption of Rb permits increased p16 expression [6, 10]. Expression of p16 is therefore up-regulated in HPV-positive cancer and frequently lost in HPV-negative tumors.

Several studies have shown that patients with HPV-associated OPSCC have a better prognosis than patients with HPV-negative tumors, with significantly decreased risk of death (40–60% reduction) and relapse (60–70% reduction) in HPV-associated tumors compared to HPV-negative tumors, when treated with multimodality therapies [7, 8, 11–13]. HPV-positive cancers also have better outcome following induction chemotherapy (IC), radiation and chemoradiation for OPSCC patients. A prospective analysis of the association of tumor HPV status and therapeutic response and survival among 96 patients with stage III/IV HNSCC of oropharynx or larynx treated with IC followed by concurrent chemoradiotherapy on the ECOG 2399 phase II trial showed that patients with HPV-ISH-positive or p16-positive tumors had significantly higher response rates (RR) after IC and after paclitaxel-based chemoradiotherapy compared with patients with HPV-negative tumors. After a median follow-up of 39.1 months, patients with HPV-associated tumors also had significantly improved overall survival (OS) and lower risks of progression than those with HPV-negative tumors [8]. In the recent E1308 phase II trial, 90 patients with HPV16 and/or p16-positive stage III-IV OPSCC received three cycles of IC with cisplatin, paclitaxel, and cetuximab, after which patients with primary-site complete clinical response (cCR) received intensity-modulated radiation therapy (IMRT) 54 Gy with weekly cetuximab, while those with less than cCR received 69.3 Gy and cetuximab. The primary end-point was two-year progression-free survival (PFS). Fifty-six patients (70%) achieved a primary-site cCR to IC and 51 patients continued to cetuximab with IMRT 54 Gy. After median follow-up of 35.4 months, two-year PFS and OS rates were 80% and 94%, respectively, for patients with primary-site cCR treated with 54 Gy of radiation (*n* = 51); and 96% and 96%, respectively, for patients with < T4, < N2c, and <10 pack-year smoking history who were treated with ≤54 Gy of radiation (*n* = 27). At 12 months, significantly fewer patients treated with a radiation dose ≤54 Gy had difficulty swallowing solids (40% v 89%; *P* = 0.011) or had impaired nutrition (10% v 44%; *P* = 0.025). The study therefore suggests that for IC responders, reduced-dose IMRT with concurrent cetuximab should be considered in favorable-risk patients with HPV-associated OPSCC since de-intensification with radiation dose reduction resulted in significantly improved swallowing and nutritional status [14]. Another biomarker analysis studied the association of HPV with clinical outcomes in recurrent or metastatic (R/M) HNSCC patients treated on two clinical trials: E1395, a phase III trial of cisplatin and paclitaxel versus cisplatin and 5-fluorouracil, and E3301, a phase II trial of irinotecan and docetaxel [15]. HPV DNA was detected by ISH and p16 status was evaluated by IHC. Sixty-four patients were analyzed for HPV ISH and 65 for p16. Eleven tumors (17%) were HPV-positive, 12 (18%) were p16-positive, whereas 52 (80%) were both HPV and p16-negative. There was significantly improved objective response rate (ORR) for HPV-positive versus HPV-negative (55% vs 19%; *P* = 0.022), and for p16-positive versus p16-negative (50% vs. 19%; *P* = 0.057) tumors. There was also improved median survival for HPV-positive versus HPV-negative patients (12.9 vs. 6.7 months; *P* = 0.014), and for p16-positive versus p16-negative patients (11.9 vs. 6.7 months; *P* = 0.027). After adjusting for other covariates, hazard ratio (HR) for OS was 2.69 (*P* = 0.048) and 2.17 (*P* = 0.10), favoring HPV-positive and p16-positive patients, respectively [15]. HPV is therefore a favorable prognostic factor in R/M HNSCC.

The predictive role of HPV status with specific therapy has been less well understood. Epidermal growth factor receptor (EGFR) inhibitors in particular have been studied in this regard. Subset analysis of the SPECTRUM phase III trial of chemotherapy with or without the anti-EGFR antibody panitumumab in R/M HNSCC suggested that p16-negative patients had benefit to addition of the human anti-EGFR antibody, panitumumab, unlike p16-positive patients [11]. However, the significance of the data has been called into question because of the limited cohort of p16-positive patients across subsites and the high rates of p16 positivity outside the oropharynx, as well as by the fact that pantitumumab has not prolonged survival in HNSCC in any trial in any line of therapy. Biomarker analysis of HPV-association conducted on the similarly designed EXTREME phase III trial of chemotherapy with or without cetuximab showed that the benefits of chemotherapy and cetuximab over chemotherapy alone appeared to be independent of HPV/p16 status. This analysis was however limited by the small number of patients with HPV-positive (5%) and p16-positive (10%) tumors [13]. A secondary analysis of the MCL-9815 (Bonner) phase III trial examined the association of HPV DNA status and p16 expression with outcomes in patients with OPSCC treated with cetuximab plus RT versus RT alone in the definitive setting [13]. Although sample sizes precluded conclusive tests of interaction in this study, the results suggest that regardless of p16 status, patient’s outcomes were improved by the addition of cetuximab to RT compared with RT alone. Interestingly, the benefit of cetuximab in the p16-positive population was more pronounced compared to the p16-negative population, with improved locoregional control (LRC) and OS with the addition of cetuximab to RT compared with RT alone in p16-positive (HPV-associated) OPSCC. The HR for LRC and OS for HPV-associated were 0.31 (95% CI; 0.11–0.88) and 0.38 (95% CI; 0.15–0.94) respectively compared to HR of 0.78 (95% CI; 0.49–1.25) and 0.93 (95% CI; 0.59–1.48) in HPV-negative patients [13].

### HPV status and p-16 in non-OPSCC

The clinical significance of p16 positivity in non-OPSCC is less clear than for OPSCC, however patients with p16-positive non-OPSCC have better outcomes than patients with p16-negative non-OPSCC, similar to findings in patients with OPSCC. In a retrospective analysis of non-OPSCC tumors from 332 patients enrolled on three RTOG studies, overall p16 expression was positive in 19.3% of the non-OPSCC tumors with the rates of p16 positivity of 14.1%, 24.2% and 19% for RTOG 0129, 0234 and 0522 studies, respectively [16]. In this study, patients with p16-positive non-OPSCC tumors had a better prognosis compared with those who were p16-negative, after adjusting for known prognostic factors including age, sex, T stage and N stage. For PFS, the adjusted HR was 0.63 (95% CI 0.42–0.95, *P* = 0.03), while for OS the adjusted HR was 0.56 (95% CI 0.35–0.89, *P* = 0.01). Comparing OPSCC and non-OPSCC patients from the same studies, p16-positive OPSCC have better survival than patients with p16-positive non-OPSCC (HR for OS of 0.48; 95% CI 0.30–0.78), but patients with p16-negative OPSCC and non-OPSCC have similar survival, even after adjustment of prognostic variables (HR for OS of 0.97; 95% CI 0.74–1.24). A recent study suggested that HNSCC associated with HPV genotypes other than HPV-16 have inferior survival_,_ and that determination of HPV genotypes in HNSCC could provide a more robust risk stratification than p16 IHC findings or HPV-16 detection alone, especially in the era of treatment de-intensification for HPV-associated HNSCC [17]. In this study, 551 HNSCC tumors from the cancer genome atlas (TCGA) were analyzed, along with corresponding patient data, looking at 179 distinct HPV genotypes. Seventy-three tumors expressed HPV transcripts, among which 61 (84%) were HPV-16 genotype and twelve (16%) were HPV-other genotypes. The study showed that three-year OS was significantly worse for the non-HPV-16 cohort (49%) compared to the HPV-16 cohort (88%), *P* = 0.003 [17]. However, the significance of the data has been called into question because 41% of HPV-other genotypes were detected outside the oropharynx, the prognostic impact of observed differences in viral gene expression found in the study remains unclear, and the clinically validated biomarker p16 was available only for one-third of HPV-other genotype cases [18]. Further prospective studies of HPV-other genotypes in OPSCC will be required before we can conclude that HPV genotype alone can serve as patient selection factor precluding treatment de-intensification.

### Plasma EBV in nasopharyngeal carcinoma

NPC is the predominant tumor type arising in the epithelial lining of the nasopharynx, and differs from other HNSCC in epidemiology, histology, natural history, and response to therapy [19]. The World Health Organization (WHO) classifies NPC into the three histopathologic types, including the keratinizing SCC subtype (WHO type I), the differentiated, non-keratinizing sub-type (WHO type II) and the undifferentiated, non-keratinizing sub-type (WHO type III) [20]. The sporadic form of NPC is most commonly the keratinizing subtype (WHO type I) while the endemic form of NPC is commonly the undifferentiated, non-keratinizing subtype (WHO type III). This endemic form is strongly associated with EBV and has a more favorable prognosis than other types [19]. The incidence of NPC demonstrates a marked geographical variation. It is rare in the United States and Western Europe, but endemic in Southern China, while intermediate-risk regions include Southeast Asia, North Africa, the Middle East, and the Arctic [19]. There is a multifactorial etiology, which to an extent explains the geographic variation of incidence. In endemic populations, risk appears to be due to an interaction of several factors including EBV infection, environmental factors such as smoking, and genetic predisposition. Smoking may be involved in the pathogenesis of NPC by causing EBV reactivation [21, 22]. A study in China showed that smoking is associated with increased risk of NPC Chinese patients with 20–40 and 40 or more pack-years vs. never smokers (OR = 1.52, 95% CI = 1.22–1.88 and OR = 1.76, 95% CI = 1.34 to 2.32, respectively; *P* < 0.001) [23]. In vitro analysis showed that exposure of cells to cigarette smoke extract promoted EBV replication, induced the expression of the immediate-early transcriptional activators *Zta* and *Rta*, and increased transcriptional expression of its lytic gene products, *BFRF3* and *gp350* [23]. In the US and Europe, NPC is more commonly associated with alcohol and tobacco usage, which are classic risk factors for other HNSCC [24].

The role of EBV as a primary etiologic agent in the pathogenesis of NPC is well established [25]. EBV DNA and EBV gene expression has been identified in precursor lesions and tumor cells. NPC cells express a specific subgroup of EBV-latent proteins, including EBNA-1 and two integral membrane proteins, LMP-1 and LMP-2, along with the BamHI-A fragment of the EBV genome. Patients with NPC also demonstrate specific immunologic responses to various gene products of EBV, particularly immunoglobulin A (IgA) antibodies directed against the EBV viral capsid antigen [25, 26]. This association of NPC with EBV infection has been harnessed to develop noninvasive diagnostic tests, some of which have been explored as clinical biomarkers. Plasma EBV DNA is currently the most reliable and accurate predictive and prognostic biomarker for NPC and has utility in diagnosis, prognosis, surveillance and assessment of response to therapy. Pre-treatment EBV DNA was a found in 96% of NPC patients in Hong Kong, and high levels of EBV DNA was associated with advanced disease, disease relapse and worse outcome [27, 28]. Elevated post-treatment EBV DNA is a strong negative prognostic factor in prospective trials of RT alone, concurrent chemoradiotherapy or IC followed by RT [29, 30]. A prospective study evaluated the use of serial plasma EBV DNA on the long-term survival of non-metastatic NPC patients treated with IMRT +/− adjunct chemotherapy by time-dependent receiver operating characteristics (TD-ROC) [31]. Baseline plasma EBV was assessed, then repeated at 8 weeks and 6 months after IMRT, after which survival outcome was analyzed. Results revealed that post-IMRT undetectable plasma EBV DNA accurately predicted almost all survival endpoints and early post-IMRT plasma EBV DNA should be regarded as a new sentinel time point to consider further intensified treatment or not after completion of chemo IMRT. NCT02135042 (NRG-HN001) is an ongoing randomized phase II/III study evaluating individualized treatment for NPC based on biomarker EBV DNA expression [32]. The study is based on two cohorts of patients with a diagnosis of stage II-IVB non-metastatic NPC and detectable pre-treatment plasma EBV DNA. In the persistently detectable plasma EBV DNA cohort (phase II), the primary objective is to determine whether substituting adjuvant CDDP and 5-FU with gemcitabine and paclitaxel will result in superior PFS. In the second cohort, the undetectable plasma EBV DNA cohort (phase III), the primary objective is to determine whether omitting adjuvant CDDP and 5-FU (observation alone in the adjuvant setting) will result in non-inferior OS compared to those patients that receive conventional treatment with adjuvant CDDP and 5-FU chemotherapy.

### Emerging/novel biomarkers

**Fig. 1:**
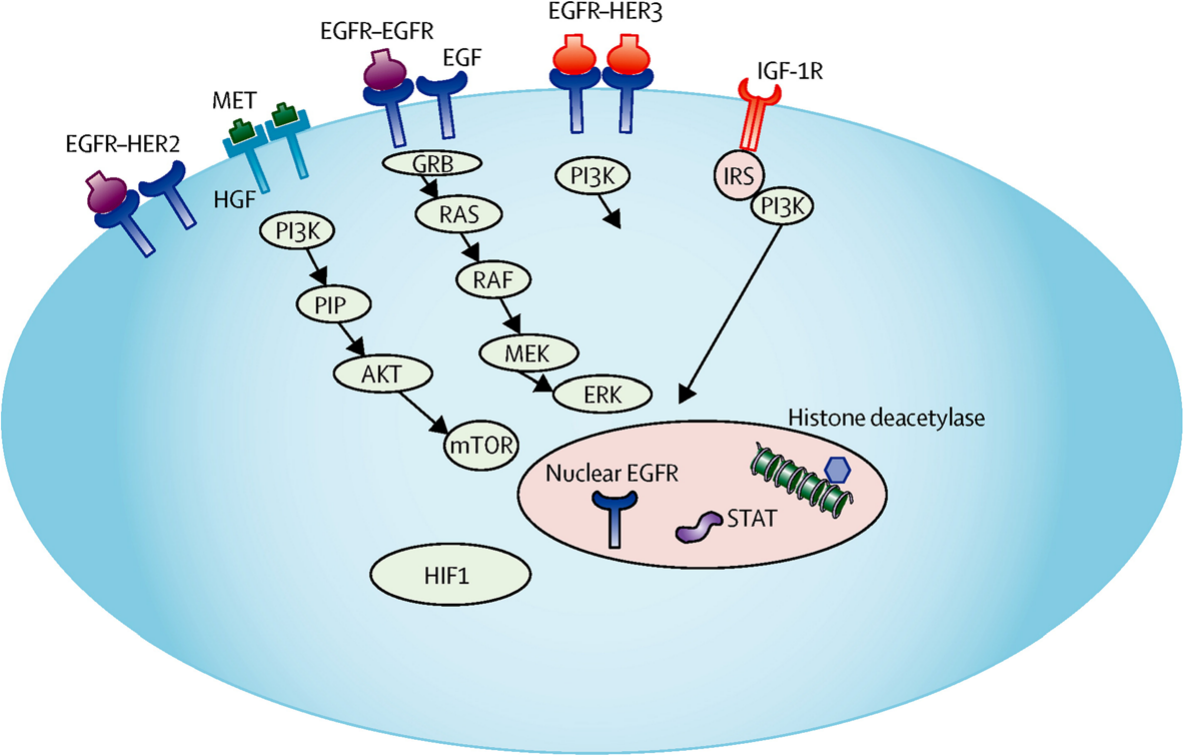
EGFR and receptor tyrosine kinase signaling in head and neck cancer. Resistance to EGFR inhibition may arise via signaling from redundant tyrosine kinases, such as HER family members, as well as downstream signaling activation. These may be important biomarkers predicting therapeutic response in head and neck cancer

The landscape of HNSCC treatment is changing with the emergence of tumor biomarkers, some of which are potential pharmacologic targets. Downstream abnormalities associated with constitutive activation and signaling of the EGFR pathway may be an important therapeutic target in HNSCC especially in HPV-negative tumors (Fig. 1).

### Targeting receptor tyrosine kinases and their signaling pathways

Dysregulation of EGFR signaling has been shown to stimulate tumor cell proliferation, inhibit apoptosis, and promote angiogenesis and metastatic spread; and aberrations of the EGFR pathway are a common feature of HNSCC and are associated with worse prognosis [33]. Based on current genome-wide sequencing data, only a few oncogenes in HNSCC are immediately targetable with drugs in clinical development. These include *EGFR, PIK3CA, FGFR, MET* and *CCND1.*

#### PI3K/MTOR pathway

Genetic aberrations of the phosphoinositide 3- kinase (PI3K) pathway are common in HNSCC [34]. Phosphatidylinositol-4, 5-biphosphate 3-kinase, catalytic subunit alpha (*PIK3CA*) encodes p110α, a catalytic subunit of PI3K and activated PI3K triggers downstream effects on transcription, protein synthesis, metabolism, proliferation and apoptosis [35]. It was shown in correlative studies from the E2303 trial of cetuximab-based induction and chemo-radiotherapy in locally advanced HNSCC that PI3K/AKT pathway activation is associated with inferior PFS and OS and may predict resistance to EGFR-targeted therapy [36]. Previous data suggested *PIK3CA* mutations in approximately 8% of HNSCC samples [37], but more recent data from TCGA study identified *PIK3CA* mutations in 21% of HNSCC samples, with 73% of the *PIK3CA* mutations localized to hotspots that promote activation [38]. HPV-negative samples were noted to have 18% *PIK3CA* mutations whereas HPV-positive samples harbored 38% *PIK3CA* mutations. Additionally, *PIK3CA* mutations and/or amplifications were observed in 37% of the HNSCC (34% of HPV-negative and 56% of HPV-positive) samples. Approximately 25% of the mutated *PIK3CA* cases displayed concurrent amplification; while additional 20% of tumors displayed focal amplification without evidence of mutation [38]. The data also suggest that there are differences in the *PIK3CA* mutation hotspots between HPV-positive and HPV-negative tumors. HPV-positive tumors were observed to have mutations in the helical domain, whereas HPV-negative tumors have mutations throughout the entire gene [38].

The PI3K inhibitor buparlisib (BKM120) is an oral pan-PI3K inhibitor that targets all four isoforms of class I PI3K. When used in combination with paclitaxel, buparlisib has demonstrated improved outcomes in patients with R/M HNSCC compared to paclitaxel alone, with a median PFS of 4.6 versus 3.5 months (HR = 0.65), a median OS of 10.4 versus 6.5 months (HR = 0.72), as well as improved ORR 39% versus 14% [39]. Data regarding PIK3CA mutational status and PTEN content were not presented, and although it is not presently known whether patient selection will be required for this therapy, it is likely that buparlisib/paclitaxel combination will emerge as a treatment option for R/M HNSCC.

#### PTEN

A common downstream abnormality associated with activation and signaling in HNSCC is loss of phosphatase and tensin homolog (PTEN) expression. PTEN is a key negative regulator of the PI3K/AKT/mTOR pathway and PTEN loss results in unrestrained signaling of this pathway [35]. There is loss of PTEN expression in about 30% of HNSCC, either via *PTEN* mutation or post-translational modification, [40–42] and this may be associated with worse outcome in HNSCC [41]. In a study on HPV-positive OPSCC, PTEN loss (assessed by FISH) was identified in 7/21 (33%) cases, suggesting PTEN loss may be independent of HPV status [43]. Another study analyzed DNA samples obtained from 252 formalin-fixed paraffin-embedded (FFPE) HNSCC tumor samples using next-generation sequencing-based (NGS) clinical assay [44]. HPV status was determined by presence of the HPV DNA sequence and corroborated with high-risk HPV ISH and p16 IHC staining in a subset of tumors. This study demonstrated *PTEN* genomic alterations (PTEN mutation or loss) in 15% of HPV-positive and 5% of HPV-negative tumors [44]. In another recent study, the expression of PTEN, p53, PIK3CA, Akt and mTOR (all evaluated by IHC) were investigated according to HPV status (evaluated by ISH) in 65 tonsillar SCC tumors. [45] This study demonstrated that total PTEN (nuclear and cytoplasmic) expression was more frequently observed in HPV-positive compared to HPV-negative tonsillar SCC cases (*P* = 0.037), with predominant PTEN distribution in the nucleus. Overall, PTEN expression was lost in 47% of tumors and preserved in 53% of tumors. PTEN was negative in 27% of HPV-positive compared to 57% of HPV-negative tumors. The study also showed a significant correlation between nuclear PTEN expression and DFS (*P* = 0.27). There was no difference in expression of p53, PI3K, Akt and mTOR between HPV-positive and HPV-negative cases [45].

In preclinical models of breast, prostate and non-small cell lung cancer, PTEN loss has been shown to be associated with cetuximab resistance [46]. Biomarker analysis of the E5397 phase III study suggested that the addition of cetuximab to cisplatin in R/M HNSCC improved PFS in PTEN high/PIK3CA wild type patients (representing the group with non-activation of PI3K pathway; *P* = 0.07) but not PTEN null/PIK3CA mutant patients (representing the group with activation of PI3K pathway; *P* = 0.6) [47]. This suggests that there may be cetuximab resistance when the PI3K pathway is activated downstream of EGFR. LUX- Head and Neck 1 studied another active EGFR inhibitor, afatinib, in patients with previously treated R/M HNSCC, demonstrating improved PFS but not significantly improved OS in this population [48]. Biomarker analysis suggests that afatinib utility could be improved with the use of biomarker patient enrichment. PTEN, p16 and HER3 status are evaluated by IHC while EGFR amplification is evaluated by FISH. Overall, the study appeared to show a more pronounced effect on outcome with afatinib vs. MTX in p16- negative, EGFR-amplified, HER3-low and PTEN-high tumors. However, the p16 data were underpowered as the sample size of p16-positive patients was small in this study. In PTEN high tumors, afatinib showed a significantly improved PFS when compared to MTX, with a median PFS of 2.9 vs. 1.4 months (HR of 0.36; 95% CI 0.16–0.81, *P* = 0.014). In HER3 low tumors, afatinib also demonstrated a significantly improved PFS compared to MTX, with a median PFS of 2.9 vs. 2.0 months (HR of 0.47; 95% CI 0.25–0.86, *P* = 0.014) [48, 49].

#### EGFR over-expression

EGFR over-expression is a negative prognostic factor after radiotherapy but has not been validated as predictive biomarker [50]. The E5397 phase III trial of cisplatin plus placebo versus cisplatin plus cetuximab for first-line treatment of R/M HNSCC suggested it might have a predictive role [47]. In this study, almost all the patients had EGFR over-expression. The RR only improved from 6% to 12% (*P* = 0.99) with addition of cetuximab in patients with very high EGFR expression (IHC 3+ in 80–100% of cells). In contrast, there was a more dramatic improvement in RR, from 12% to 41% (*P* = 0.03), with addition of cetuximab in patients with low to moderate EGFR expression (IHC 3+ in 0–79% of cells). Although, the interaction between EGFR and treatment group was not found to be statistically significant in a logistic regression analysis of response, there appeared to be reduced benefit of cetuximab in patients with very high EGFR expression compared to patients with low to moderate EGFR expression. Based on this study, highest EGFR expression intensity and density appear to define a group, representing about a third of the cohort, with lesser sensitivity to EGFR inhibition.

#### FGFR

The fibroblast growth factor receptor (FGFR) signaling pathway plays a role in cellular differentiation, proliferation, apoptosis, migration, angiogenesis and wound repair. FGF binding to members of this family of trans-membrane tyrosine kinase receptors with four members (FGFR1–4) leads to FGFR dimerization and activation of downstream signaling pathways including MAPK, PI3K/AKT/MTOR, and STAT pathways [51]. Activating mutations, amplification and translocation resulting in fusion genes involving these receptors have been reported in many cancers, including HNSCC. FGFR1 amplification or mutation is seen in 10% of HPV-negative HNSCC, while FGFR3 mutations or fusions occur in 11% of HPV-positive HNSCC [38]. FGFR inhibition has been extensively studied in HNSCC and targeting FGFRs is a promising therapeutic strategy in HNSCC. The FGFR inhibitor PD173074 was shown to reduce cell proliferation and increase cell apoptosis in HNSCC in vitro and in vivo [52]. Selective FGFR inhibitors are being evaluated in several cancers harboring FGFR amplification and mutation. BGJ398 is a pan FGFR kinase inhibitor that has been tested in a phase I dose escalation study in patients with advanced solid malignancies harboring either FGFR1 or FGFR2 amplification or FGFR3 mutations (NCT01004224) [53]. An ongoing JNJ-42756493 phase I study includes efforts to optimize dose and schedule and to analyze biomarkers. Expansion cohorts are currently enrolling patients with FGFR-aberrant tumors, including HNSCC (NCT01703481) [54].

#### Cyclin D1

Cyclin D1 is encoded by *CCND1* and is a cell-cycle protein that regulates the key G1-to-S phase transition through formation of complexes with CDKs, such as CDK 4 and 6. The cyclin D1-CDK4/6 complex phosphorylates Rb on tyrosine residue 356 (phospho-^T356^), inactivating Rb and releasing the inhibition of cell cycle progression by Rb [55]. Alterations in cyclin D-CDK4/6-Rb pathway such as *CCND1* amplification can lead to uncontrolled tumor cell proliferation via sustained activation of CDK 4/6 and inactivation of Rb [55, 56]. In a recent TCGA study, 28% of HNSCC had *CCND1* amplification, with 77/243 (32%) in HPV-negative and 2/36 (6%) in HPV-positive samples [57]. Over-expression of cyclin D1 and amplification of *CCND1* in HNSCC are associated with poor prognosis and resistance to cisplatin and EGFR inhibition [58, 59]. Targeting of cyclin D1 is not currently feasible, though inhibition of its binding partners CDK4 and/or CDK6, might have a future role in patients with *CCND1* amplification. EGFR activity has been shown to regulate cell-cycle progression via ERK1/2-dependent induction of cyclin D1 [55]. A recent study investigated EGFR and HER2 expression in the context of Rb, phospho-^T356^ Rb, cyclin D1, and CDK6 in in 99 HPV-negative HNSCC patient samples and correlated this with clinical data [60]. The study demonstrated that Rb inactivation, reflected by phosphorylation of Rb, inversely correlated with expression of EGFR in HNSCC samples. Stratification of high EGFR expressors by expression levels of cyclin D1, CDK6, or the cyclin D1/CDK6-regulatory protein p16 (CDKN2A) identified groups with significant survival differences, consistent with prior studies that demonstrated improved survival in HNSCC with low levels of cyclin D1 and in those with low phosho-^T356^ Rb [61, 62]. In this study, simultaneous inhibition of Rb phosphorylation with the CDK4/6 inhibitor, palbociclib, and of EGFR activity with dual tyrosine kinase inhibitors (TKI), lapatinib or afatinib, was also performed [60]. These drug combinations showed synergistic inhibitory effects on the proliferation of HNSCC cells, suggesting that combinations of CDK and EGFR inhibitors may be particularly useful in EGFR and phosphorylated Rb-expressing or cyclin D1/CDK6-overexpressing HPV-negative HNSCC. Combined consideration of phosho-^T356^ Rb status and EGFR expression may therefore be useful as predictive biomarkers in this context and should be explored further as predictive biomarkers to select patients for therapy with EGFR/HER2 and/or CDK inhibitors.

#### C-MET

Hepatocyte growth factor receptor (HGFR) or c-MET is encoded by the *MET* gene and it is a RTC associated with enhanced migration, invasion and angiogenesis when overexpressed in cancer. Although considerable evidence implicates the MET-HGF axis as a therapeutic target in HNSCC [63], appropriate assays to detect aberrations in MET and its ligand HGF are lacking and further investigation is warranted.

### Immune checkpoint related biomarkers

PD-L1, PD-L2 and IFN-gamma are potential immune biomarkers shown to correlate with response to immunotherapy in R/M HNSCC [64]. Pembrolizumab has shown promising efficacy in R/M HNSCC in the phase I KEYNOTE-012 study. In this study, analysis of PD-L1 showed an increase in ORR between PD-L1 positive versus PD-L1 negative tumors (*P* = 0.23) when both tumor and stromal cells were used to score PD-L1 [65]. Assessing RNA expression of IFN-gamma related genes using a six-gene signature (*CXCL9*, *CXCL10*, *IDO1*, *IFNG*, *HLA-DRA* and *STAT1*) identified in a melanoma cohort in the KEYNOTE-001 study [66], showed that all six IFN-gamma related genes had significantly higher mean expression values in pembrolizumab-responders compared to non-responders [65]. Exploratory analyses suggest that PD-L2 and IFN-gamma signature may be associated with clinical response in pembrolizumab and may offer additional strategies to improve prediction of response. In the recent Phase III CheckMate-141 study, nivolumab, an anti-PD-1 monoclonal antibody, was shown to improve OS in patients with platinum-refractory R/M HNSCC compared to single agent therapy of the investigator’s choice, consisting of MTX, docetaxel or cetuximab [64]. Patients with PD-L1 expression >1% had significantly longer median OS (8.7 months vs. 4.6 months, HR: 0.55, 95% CI: 0.36–0.83) with nivolumab than with investigator’s choice.

### Tumor suppressor abnormalities

#### TP53

*TP53* is the most commonly mutated gene in HNSCC and is present in about 50–80% of HNSCC [67, 68]. Disruptive *TP53* mutation in tumor DNA has been shown to correlate with worse prognosis after surgical treatment of HNSCC [68]. The p53 protein is a transcription factor and tumor suppressor protein encoded by *TP53*. Loss of p53 function occurs in more than 90% of HNSCC through loss of heterozygosity, interaction with HPV viral oncoprotein E6 or increased expression of MDM2 (seen in about 5% of HNSCC and promotes rapid degradation of p53 protein) [37, 69]. An inverse relationship between the presence of a *TP53* mutation and the presence of HPV DNA in OPSCC may be due to the contribution of high-risk HPV infection, in which p53 is rapidly degraded after interacting with E6 [68, 70, 71]. Inhibition of WEE1, a G2-M cell-cycle regulator, can render synthetic lethality in *TP53*-mutant tumors because cells without functional p53 lack an effective G1 checkpoint and rely heavily on the G2 checkpoint regulators, such as WEE1, resulting in increased sensitivity of *TP53*-mutant cells to WEE1 inhibitors. Thus, *TP53* mutations need to be further investigated as a predictive biomarkers and therapeutic target in HNSCC [72].

#### Notch

The Notch pathway consists of four receptors, Notch 1–4. Activation of the Notch pathway leads to different effects in different cell types. *NOTCH-1* is believed to play a role in regulating normal cell differentiation and has dual functions with both oncogenic and tumor suppressor activity. In epithelial tissue, including HNSCC, *NOTCH-1* appears to act as a tumor suppressor gene [37, 73]. Two independent whole exome sequencing studies report *NOTCH1* mutations in about 14% and 15% of HNSCC respectively [37, 74], and these studies hypothesize that *NOTCH1* functions as a tumor suppressor in HNSCC based on its mutational characteristics. Evidence also suggest that the majority of the mutations identified in exome sequencing are likely inactivating or loss of function mutations that affect the EGF-like ligand binding domain or the *NOTCH* intracellular domain [37, 73]. In one of the studies that examined 32 patients with mostly pre-treated HNSCC tumors, *NOTCH1* was the second most frequently mutated gene found, next to *TP53,* with alterations present in 15% of patients [74]. In this study, 28 *NOTCH1* mutations were identified and nearly 40% of these *NOTCH1* mutations were predicted to truncate the gene product, again suggesting that *NOTCH1* may function as a tumor suppressor gene rather than an oncogene in this tumor type. Other reports also suggest that a subset of HNSCC may have activating *NOTCH1* mutations [75], with overexpression of downstream Notch effectors noted in 32% of HNSCC evaluated for DNA-copy number, methylation and gene expression of the 47 Notch signaling pathway genes. This indicates that the Notch1 pathway could be a potential therapeutic target in a subset of HNSCC. Therapeutic targeting of *NOTCH-1* in HNSCC remains an evolving field.

### Tumor hypoxia as predictive biomarker in HNSCC

A hypoxic microenvironment is a common feature in HNSCC, and contributes to the development of tumor aggression and metastasis, playing a key role in radio-resistance, chemo-resistance, and poor prognosis. Acute hypoxic stress leads to the development of an aggressive cancer phenotype with high metastatic rate, resistance to therapeutic agents, and higher tumor recurrence rates [76]. This is mostly mediated by hypoxia inducible factor-1- alpha (HIF-1*α*), which is over-expressed in HNSCC*,* and plays a central role in hypoxia-induced therapeutic resistance in HNSCC through its role in initiating angiogenesis and regulating cellular metabolism to overcome hypoxia [77]. Therefore, HIF-1*α* and its downstream proteins are potential predictive biomarkers and therapeutic targets in HNSCC. Strategies to overcome hypoxia-induced therapeutic resistance include the use of hypoxic cell cytotoxins like tirapazamine (TPZ), enhancing oxygen delivery using hyperbaric oxygen, and use of hypoxic cell radiosensitizers. TPZ is reduced to a reactive radical when exposed to hypoxic conditions, leading to single- and double-strand DNA breaks. In contrast, this reactive radical is oxidized to the inert parent compound in normal oxygen tension. A prospective trial [78] evaluated the combination of TPZ with cisplatin and radiation in advanced HNSCC using [18]F fluoromisonidazole PET imaging as a biomarker to measure hypoxia levels. The study demonstrated that hypoxia levels decreased with treatment and showed that combination of TPZ with cisplatin and radiotherapy led to durable clinical responses with three-year EFS of 69%, a three-year local PFS of 88%, and a three-year OS of 69%. In another phase II trial, it was demonstrated that patients treated with TPZ, cisplatin and radiation had higher three-year EFS and three-year locoregional PFS than patients treated with cisplatin, fluorouracil and radiation, with less radiation-induced toxicities [79]. A prospective study assessed the efficacy of misonidazole, a hypoxic cell radiosensitizer, in 626 patients with pharynx and larynx carcinoma and showed that patients with pharyngeal carcinoma treated with misonidazole exhibited a significantly better control disease rate than patients treated with placebo [80]. However, the clinical use of misonidazole is limited because it caused significant peripheral neuropathy in 26% of the patients. Another phase III clinical study assessed the efficacy and tolerance of nimorazole in combination with primary radiotherapy in 422 patients with pharynx and supraglottic larynx carcinoma, and showed that patients treated with nimorazole displayed a better locoregional control and OS than patients that received placebo [81]. These findings suggest that hypoxia biomarkers have the potential to predict response to hypoxic-cell radiosensitizers or cytotoxins. Although attempts to target tissue hypoxia, including TPZ, have not been successful in large phase III trials, patient selection via biomarkers of hypoxia was not employed in these trials and would merit further exploration.

### Tumor hypoxia and interleukin-8 (IL-8)

Attempts have been made to identify molecular predictors for hypoxia-targeted therapy. IL-8 has been shown to be an independent prognostic factor in HNSCC patients irrespective of treatment. A randomized study investigated the prognostic and predictive significance of IL-8 and hepatocyte growth factor (HGF or scatter factor), a hypoxia- induced secretory protein that binds c-MET and regulates IL-8 expression, on the efficacy of TPZ [82]. Four hundred and ninety-eight patients with Stage III–IV HNSCC were randomized to receive radiotherapy with cisplatin (control arm) or cisplatin plus TPZ (treatment arm). Eligibility criteria included plasma sample availability for HGF, IL-8 assay by ELISA and no major radiation deviations. Analyses included adjustment for major prognostic factors. p16 staining was performed on available tumors. Findings suggest that IL-8 is an independent prognostic factor irrespective of treatment and that there is an interaction between treatment arm and HGF level. Elevated IL-8 level was associated with worse OS irrespective of treatment. Elevated HGF was associated with significantly worse OS in the control but not in the TPZ/CIS arm (*P* = 0.053). Similar trends were observed in analyses restricted to p16-negative patients. Four subgroups defined by high and low HGF/IL-8 levels were examined for TPZ effect and TPZ/CIS appeared to be beneficial for patients with high HGF and IL-8, but adverse for low HGF and high IL-8. This highlights the complexity of hypoxia targeting in unselected patients.

### Futuristic biomarkers

With advancements in digital genomic technologies, such as digital PCR and BEAMing, reliable detection of circulating DNA from clinical specimens has become feasible, and is a potential future predictive biomarker in HNSCC therapy.

### Liquid biopsies

Evaluation of DNA aberrations in blood samples can be quite beneficial as it can be a challenge to obtain tumor DNA in clinical settings. Highly sensitive and specific assays are required to detect mutant DNA fragments in the blood. With advancements in digital genomic technologies, such as digital PCR, tagged-amplicon deep sequencing, pyrophosphorolysis-activated polymerization, and BEAMing, reliable detection of circulating DNA from clinical specimens has become feasible [83]. DNA from blood can be obtained by two methods, either as circulating tumor DNA (ctDNA) or from circulating tumor cells (CTC).

A recent study used digital PCR–based technologies to evaluate the ability of ctDNA to detect tumors in 640 patients with various localized and metastatic cancer types, including HNSCC [84]. ctDNA was detectable in more than 75% of patients with advanced HNSCC, and was often present in patients without detectable circulating tumor cells, suggesting that these two biomarkers are distinct entities. Using liquid biopsies, it has been shown that RAS mutations may account for acquired resistance to EGFR-targeting in a substantial proportion of HNSCC patients, even though these tumors are rarely mutated at baseline. A recent study analyzed the activating RAS mutations in tumor tissue of cetuximab-naive HNSCC patients by NGS and compared this with liquid biopsies taken during and after cetuximab/platinum/5-fluorouracil treatment [85]. Baseline data showed that tumors of cetuximab-naive patients were mostly unmutated, except for HRAS mutations in 4.3% of patients. Liquid biopsies revealed acquired KRAS, NRAS or HRAS mutations in more than one-third of patients after cetuximab exposure. Almost half of patients with on-treatment disease progression showed acquired RAS mutations, while no RAS mutations were found in the non-progressive subset of patients, indicating that acquisition of RAS mutant clones correlated significantly with clinical resistance to EGFR-inhibition. These novel assays can be applied in the early detection of cancer, surveillance after treatment, early identification of resistance to targeted agents, and to explore mechanisms of resistance without invasive tissue sampling.

### Genomic profiling

Rapid tumor profiling with sequencing of panels of several hundred cancer relevant genes is now commercially available for use in clinical practice. The relevance of this approach to management of HNSCC has not been demonstrated, given the predominance of mutations in tumor suppressor genes. A recent study compared the genomic profile of the HNSCC tumors obtained through routine clinical practice with sequencing data from frozen tumors in TCGA and University of Chicago public datasets studied in research setting [44], and the findings suggest that the selected gene analysis using FFPE tumors obtained through clinical practice yield comparable assessment of genomic alterations to frozen tumors, demonstrating the feasibility of comprehensive genomic profiling in a clinical setting. However, the clinical significance of these genomic alterations requires further investigation through application of these genomic profiles as integral biomarkers in clinical trials.

### MicroRNAs

MicroRNAs (miRNA) are a family of small, non-coding, endogenously synthesized, single-strand RNAs which are responsible for post-transcriptional regulation of mRNA expression, and have been shown to play an important role in cellular differentiation, proliferation, apoptosis, and carcinogenesis [86]. MiRNAs can be accurately measured in plasma and are potential non-invasive biomarkers for early detection of HNSCC. They are also one of the promising candidates for development of development of novel and therapeutic approaches in HNSCC. However, studies evaluating the diagnostic accuracy of miRNAs in HNSCC detection have been conflicting and inconclusive and miRNAs have not been proven to play a definite role in prognosis or predicting response to therapy in HNSCC [87].

### HNSCC biomarkers and racial disparities

There appear to be racial disparities, not only in the incidence and outcome of HNSCC, but also in the role of biomarkers in HNSCC. Many biomarker studies in HNSCC involve mostly Caucasian populations and it remains unclear if these biomarkers are applicable to non-Caucasian populations. No biomarker till date has been specifically validated in African American or other minority populations in the United States. Many prior studies suggest higher rates of HPV- associated OPSCC among Caucasians than AA [88, 89] but that may in part be due to the fact that majority of studies on HPV-associated OPSCC have been reported in Caucasian patients, with paucity of data in African American (AA) cohorts. A recent study examined the prevalence and outcomes of HPV-associated OPSCC in an AA cohort and demonstrated that HPV OPSCC is strongly present in this AA cohort. Interestingly, the study also identified an unexpectedly frequent molecular subtype in this AA cohort, HPV-positive/p16-negative tumors, with demonstrated worse outcomes than HPV-positive/p16-positive OPSCC [90]. Therefore, given these disparities, larger studies evaluating specific biomarkers in HNSCC are warranted in non-Caucasian populations.

## Conclusion

In this era of individualized medicine and biomarker-driven cancer therapy, it is important to explore robust biomarker data and incorporate them in patient selection for HNSCC therapy. We have well established prognostic biomarkers in clinical practice; however, we need to direct efforts towards development and implementation of predictive biomarkers that will aid patient selection for specific HNSCC therapies. The current standard therapies for HNSCC are either too toxic or have low response rates, and are thus not beneficial to all patients. The emphasis should be to improve patient survival and reduce treatment-related toxicity through the identification predictive biomarkers, in addition to development of specific therapies targeting these biomarkers. In patients with poor prognosis, we need to develop strategies to prevent and control recurrence and distant metastasis*.* A variety of predictive biomarkers have been discovered and are already utilized in clinical practice, while many more are being explored as therapeutic targets. Moving forward, it will be necessary for clinicians to educate themselves in order to understand basic technologies used in biomarker studies. Each biomarker needs to be critically assessed and standardized prior to application to patient care. Currently, there is no validated biomarker for minority populations in current clinical practice. Biomarkers that specifically target non- white populations should also be an area of future research as these groups of patients may be under-represented in large research studies*.* Unfortunately, although the technology and science are available, the clinical research, health-care policy and insurance policy are lagging behind, limiting the implementation of these emerging biomarkers. Nevertheless, we are optimistic that the goal of delivering individualized cancer therapy for patients with HNSCC is within our reach.

## Abbreviations

cCR: Complete clinical response
CDK: Cyclin dependent kinase
CTC: Circulating tumor cells
ctDNA: Circulating tumor DNA
DNA: Deoxyribonucleic acid
EBV: Epstein Barr virus
EGFR: Epidermal growth factor receptor
FFPE: Formalin-fixed paraffin-embedded
FGFR: Fibroblast growth factor receptor
HGFR: Hepatocyte growth factor receptor (HGFR)
HIF-1*α*: Hypoxia inducible factor-1- alpha
HNSCC: Head and neck squamous cell cancer
HPV: Human papilloma virus
HR: Hazard ratio
IC: Induction chemotherapy
Ig: Immunoglobulin
IHC: Immunohistochemistry
IMRT: Intensity modulated radiation therapy
LRC: Locoregional control
NCI: National Cancer Institute
NGS: Next-generation sequencing-based
NPC: Nasopharyngeal cancer
OPSCC: Oropharyngeal squamous cell cancer
OS: Overall survival
PFS: Progression free survival
PI3K: Phosphoinositide 3- kinase
*PIK3CA*: Phosphatidylinositol-4, 5-biphosphate 3-kinase, catalytic subunit alpha
PTEN: Phosphatase and tensin homolog
R/M: Recurrent/metastatic
Rb: Retinoblastoma
RR: Response rate
TD-ROC: Time-dependent receiver operator characteristics
TKI: Tyrosine kinase inhibitors
TPZ: Tiparazamine
WHO: World Health Organization
